# Epidemiology, risk factors, and vaccine effectiveness for SARS-CoV-2 infection among healthcare workers during the omicron pandemic in Shanghai, China

**DOI:** 10.1016/j.heliyon.2024.e32182

**Published:** 2024-05-29

**Authors:** Dan Wang, Dan Zhu, Min Xia, Xiaoying Wang, Ni Zou

**Affiliations:** Department of Infection Prevention and Control, Shanghai General Hospital, Shanghai Jiao Tong University School of Medicine, Shanghai, China

**Keywords:** COVID-19, SARS-CoV-2, Omicron, Healthcare worker, Epidemiology, Risk factor, Vaccine effectiveness

## Abstract

**Background:**

The COVID-19 pandemic has exposed healthcare workers (HCWs) to serious risk of infection. The aims of our study were to investigate the epidemiological characteristics and risk factors of SARS-CoV-2 infection among HCWs, and evaluate the vaccine effectiveness (VE) during the Omicron pandemic in Shanghai, China.

**Methods:**

Active surveillance of COVID-19 was performed among HCWs who worked in Shanghai General Hospital from December 2022 to January 2023. A case-control study was conducted by questionnaire survey to analyse the infection-related risk factors. A retrospective cohort study was explored to evaluate VE against primary infection.

**Results:**

During the Omicron outbreak, 2,008 of 2,460 (81.6%) HCWs were infected with SARS-CoV-2. The infection rate was higher in women, younger age groups, nurses and medical technicians. Among the 1,742 participants in the questionnaire, 1,463 (84.0%) were tested positive, and 95.1% of them developed symptoms. Most of the infections (53.0%) were acquired outside the hospital. The risk factors associated with higher odds of infection were working in the emergency department (aOR 3.77, 95% CI 1.69–8.38) and medical examination area (aOR 2.47, 95% CI 1.10–5.51). The protective factors associated with lower odds of infection were previous infection with SARS-CoV-2 (aOR 0.01, 95% CI 0–0.07) and receiving four doses of vaccines (aOR 0.40, 95% CI 0.17–0.97). For frontline HCWs, those who had oral-nasal exposure to coworkers were more likely to be infected (aOR 1.74, 95% CI 1.21–2.51). In VE analysis, the risk of primary infection was lower in HCWs who received the emergency heterologous booster (the fourth dose) during the epidemic (aHR 0.25, 95% CI 0.15–0.40), resulting in an adjusted-VE of 75.1%.

**Conclusions:**

In response to future pandemic, it is important for public health policies to aim at protecting HCWs through risk-differentiated infection control measures, strengthening personal protection and recommending vaccination to vulnerable individuals before the arrival of Omicron wave.

## Highlights

• SARS-CoV-2 infection rate is higher in younger age groups, nurses and medical technicians.
• Infections are predominantly acquired outside the hospital.
• Working in the emergency department and medical examination area are associated with higher odds of infection.
• Frontline healthcare workers with oral-nasal exposure to coworkers are more likely to be infected.
• Emergency heterologous booster vaccination and previous infection with SARS-CoV-2 are protective factors against infection.

## 1 Introduction

The coronavirus disease 2019 (COVID-19) caused by the transmission of severe acute respiratory syndrome coronavirus 2 (SARS-CoV-2) created a global public health crisis in late 2019 [1]. The Omicron variant that appeared in November 2021 has become the predominant strain and caused many waves of the COVID-19 pandemic worldwide. This is attributable to the variant's enhanced transmission and immune escape abilities compared with other variants of concern [2,3]. According to the latest data from the World Health Organization, by March 2023, over 765 million confirmed cases and over 6.9 million deaths have been reported globally [4]. It was estimated that almost half of the world population had been infected with the omicron variant [5].

Healthcare workers (HCWs) are at increased risk of developing COVID-19 because of their close contact with highly infectious patients [6]. Earlier studies have reported that HCWs are at 10 times more risk of contracting COVID-19 than the general population [7,8]. Many countries have reported substantial numbers of infections even deaths among HCWs during the early waves of the pandemic, bringing a considerable strain on medical system [9]. Understanding the factors exposing HCWs to a differentially high-risk of COVID-19 infection is important for healthcare policy-makers to implement appropriate public health interventions to protect them against infection [7].

Since the COVID-19 outbreak in Wuhan at the end of 2019, the Chinese government has made significant progress in curbing the epidemic by implementing a series of strict public health interventions. These interventions include social distancing, universal use of facemasks, intensive screening for SARS-CoV-2 infection, treatment of positive patients in designated hospitals, centralised quarantine of close contacts, lock-down of epidemic areas, and mandatory universal vaccination campaign prioritising populations at high risk of infection, such as HCWs. As of 20 January 2023, partial and complete vaccination rates in the general population reached 92.9% and 90.5%, respectively, even higher among HCWs because they were prioritised for vaccination [10].

The Omicron variant first triggered an outbreak in Shanghai in the spring of 2022, when fewer HCWs were infected because of the implementation of strict public health measures under China's dynamic zero-COVID policy and zero-infection targeted in HCWs. In consideration of the high COVID-19 vaccination coverage in China [11], along with the Omicron variant's relatively high transmissibility and low virulence compared with previous strains, the Chinese government relaxed prevention and control strategies in early December 2022 [12]. Since then, the Omicron variant rapidly spread nationwide, and Shanghai experienced the second wave of COVID-19 in mid-December 2022. HCWs were exposed to an unprecedented high risk of infection, not only from the community, but also from the workplace. They experienced a heavy workload from a surge in fever clinic visits as well as with a large number of patients from the general outpatient settings and inpatient wards whose positive and negative statuses mixed. Before the prevention policy was relaxed, a negative nucleic acid certificate was required for general outpatient and inpatient visits. The Shanghai municipal government launched an emergency COVID-19 booster vaccination program among HCWs in many public hospitals in December 2022 during the early stage of the pandemic. However, less is known about the impact of the Omicron pandemic on HCWs and the vaccine effectiveness (VE) in China.

Therefore, the primary aims of our study were to investigate the epidemiological and clinical characteristics of SARS-CoV-2 infection among HCWs and evaluate the occupational risk factors associated with infection during the second wave of the Omicron pandemic in Shanghai, China. The secondary aim was to estimate VE in this ideal population of HCWs with high COVID-19 vaccination rate and daily screening for SARS-CoV-2 infection. In response to future pandemic waves, this study is crucial to provide evidence for hospital decision-makers to establish risk-differentiated strategies for infection prevention and control (IPC) and develop vaccination schedules, to reduce the risk of SARS-CoV-2 infection among HCWs.

## 2 Materials and methods

### 2.1 Study setting

The study was performed at the Shanghai General Hospital (SGH), the only tertiary A-level general hospital with 975 beds, in Shanghai's southwest region (Songjiang District). During the Omicron pandemic from 1 December 2022 to 31 January 2023, SGH had 2,460 employees, and received accepted COVID-19 patients not only in the designated areas of fever clinics and isolation wards, but also in the general outpatient setting and inpatient ward. In response to the pandemic, SGH implemented a series of enhanced IPC measures to prevent COVID-19 transmission among HCWs: (1) strengthening active surveillance of COVID-19 by mandatory daily screening for SARS-CoV-2 by all staff using reverse transcription-polymerase chain reaction (RT-PCR) or rapid antigen test (RAT), (2) adopting a hierarchical personal protection strategy depending on the infection risk in different areas of the hospital, and (3) organising emergency booster vaccination.

### 2.2 Study design

Active surveillance of COVID-19 was performed among HCWs who worked in SGH to understand the epidemic situation of SARS-CoV-2 infection from 1 December 2022 to 31 January 2023. A questionnaire was administered to HCWs at the end of the outbreak to retrospectively investigate their demographics, infection status, vaccination status, and occupational risk factors. A test-negative case-control study was conducted among the respondents to analyse the risk factors associated with SARS-CoV-2 infection. A retrospective cohort study was explored to evaluate the effectiveness of COVID-19 vaccines in preventing primary SARS-CoV-2 infections.

### 2.3 Study population and case definition

The study population consisted of HCWs who worked in SGH during the study period, including nurses, doctors, medical technicians, administrators and support staff such as cleaners, nursing workers, security guards and delivery personnel. The cases were HCWs with SARS-CoV-2 infection confirmed by either RT-PCR or RAT positivity, according to the diagnostic criteria in the Scheme for Diagnosis and Treatment of COVID-19 (10th Trial Edition) published by the National Health Commission of China on 5 January 2023 [13]. HCWs having COVID-19 related symptoms without evidence of being RT-PCR or RAT positive were excluded. The controls were HCWs without any confirmed evidence of SARS-CoV-2 infection.

### 2.4 COVID-19 surveillance

During the Omicron period, intensive SARS-CoV-2 screening by RT-PCR was mandatory among HCWs, weekly from November 2021 to March 2022, and daily from April to December 2022. During the study period, infections were actively monitored by daily mandatory RT-PCR or RAT testing for SARS-CoV-2 among all HCWs before starting their workday. Anyone who tested positive was required to upload the test report to the employee COVID-19 reporting system through the personal Enterprise WeChat. The Department of Infection Prevention and Control checked the test results daily to ensure all staff members were tested. Infection status data were collected from the PCR laboratory and employee COVID-19 reporting system.

### 2.5 Questionnaire

An online survey was conducted among all HCWs at the end of the outbreak from 31 January to 6 February 2023. Informed consent was obtained from the HCWs. A uniform questionnaire was conducted through an online survey platform (https://www.wjx.cn). Participants were required to furnish information including demographics (sex, age, occupation, education), work category (frontline or second-line), work site, underlying disease, previous infection with SARS-CoV-2 and the infection time, vaccination status (number of doses of COVID-19 vaccine, vaccine type, date of each dose), infection status (diagnosis date and method). Infected HCWs were also required to provide infection-related information, including symptoms, treatment, progress, and recovery, and contact history in the past 14 days before infection diagnosis (whether they had contact with positive colleagues or patients, contaminated surroundings in the hospital, or positive family members or friends outside the hospital). Frontline HCWs with patient-facing tasks were required to provide information regarding patient contact and personal protection while on duty.

### 2.6 Sampling and detection

Real-time fluorescence quantitative RT-PCR for SARS-CoV-2 was performed with throat swabs to detect the ORF1ab and N gene, using the Novel Coronavirus 2019-nCoV Nucleic Acid Detection Kit (fluorescent PCR method) (Shanghai Biogerm Medical Technology Co., Ltd., Shanghai, China). The positive results were confirmed by the presence of two positive targets in one specimen or one positive target in both specimens. RAT was performed with nasal swabs using the Novel Coronavirus (2019-nCoV) Rapid Antigen Test (colloidal gold method) (Zhejiang Orient Gene Biotech Co., Ltd., Huzhou, China). All the tests were performed according to the product instruction.

### 2.7 Vaccination schedule

In our study, three rounds of COVID-19 vaccination were organised centrally among HCWs in SGH over the past 2 years: the first and second vaccination doses (mandatory fundamental immunisation) from December 2020 to April 2021, the third vaccination dose (first booster) in October 2021, and the fourth vaccination dose (emergency booster) during the Omicron wave in December 2022. The first two rounds of vaccination were inactivated vaccines, including Sinopharm/BIBP COVID-19, Sinopharm/WIBP COVID-19, and Sinovac-CoronaVac. The emergency booster vaccination administered in December 2022 was an adenovirus vector vaccine called the Cansino Ad5-nCoV-S COVID-19 vaccine.

### 2.8 Evaluation of vaccine effectiveness

Based on the questionnaire responses, we excluded individuals with previous SARS-CoV-2 infection to form a retrospective cohort and evaluate the effectiveness of the COVID-19 vaccine (especially the emergency booster) against primary SARS-CoV-2 infection. HCWs were followed up from 1 December 2022 until SARS-CoV-2 infection or (if never infected) until 31 January 2023. Those who received vaccination during the study period were followed up from the date of vaccination. HCWs who developed infections within 7 days after two or more vaccine doses or 14 days after the first dose were assigned the respective doses.

The vaccination status was categorised based on the number of doses of vaccine received over 7 days (over 14 days if only one dose) before infection (0, 1, 2, 3 or 4 doses), the vaccination course (unvaccinated, partially vaccinated, fully vaccinated or boosted), the vaccine type of last dose (inactivated or adenovirus vector vaccine), and the time since the last dose of vaccine through the date of infection diagnosis (<6 months, ≥6 months, during the epidemic). Unvaccinated was never receiving any COVID-19 vaccine, partially vaccinated was receiving only one dose of inactivated vaccine, fully vaccinated was receiving two doses of inactivated vaccines or one dose of adenovirus vector vaccine, and boosted was completing full vaccination plus one or two booster doses of either inactivated or adenovirus vector vaccine. Booster vaccinations were divided into homologous and heterologous boosters according to whether the type of booster was consistent with previous doses.

### 2.9 Statistical analysis

The data was analysed using IBM SPSS (version 26.0) Statistics (International Business Machines Corporation, Armonk, NY, USA). Categorical variables were presented as frequency and percentage, and continuous variables were described as mean ± standard deviation after checking for normality of distribution. The *t*-test was used for comparison of normally distributed continuous variables and the chi-squared test was used for comparison of categorical variables. *P* value*s* of <0.05 were considered statistically significant.

To analyse the risk factors, we used univariate and multivariate logistic regression to determine the crude and adjusted associations of the risk factor variables with SARS-CoV-2 infection. We reported the results as crude odds ratios (ORs), adjusted odds ratios (aORs), and 95% confidence intervals (CIs).

To analyse vaccine effectiveness, a univariate Cox proportional hazard regression model was fitted to investigate the incidence of primary SARS-CoV-2 infection by the number of vaccine doses and the time since the last dose through infection. The same covariates as the logistic regression model were included in the multivariate Cox proportional hazard regression model. The results were reported as crude hazard ratios (HRs) and adjusted HRs (aHRs) with 95% CIs. The vaccine effectiveness and corresponding 95% CI were estimated by (1-aHR) × 100%.

## 3 Results

### 3.1 Epidemiological characteristics of SARS-CoV-2 infection

**Fig. 1:**
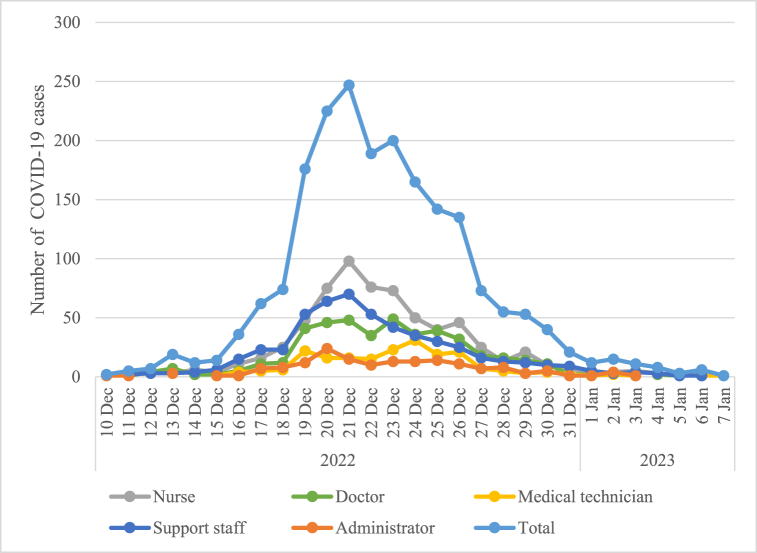
Time distribution of COVID-19 cases for different occupations based on surveillance data.

The Omicron outbreak started on 10 December 2022 and ended on 7 January 2023, lasting for 28 days, with a peak occurring between 20 December and 21 December 2022. The time distribution of COVID-19 cases is shown in Fig. 1. Among the 2,460 HCWs, 2,008 (81.6%) were infected with SARS-CoV-2. The infection rate was higher in women than in men (84.4% vs 76.5%), and decreased with age (*P* < 0.001). The infection rates varied across different occupational groups (*P* < 0.001), with the highest in nurses (88.6%), followed by medical technicians (85.7%) and the lowest in administrators (74.5%) (Table 1).

**Table 1:** SARS-CoV-2 infection rate in HCWs stratified by demographics.

Demographics	Total HCWs N = 2460	SARS-CoV-2 infected HCWs N = 2008	Infection rate (%)	Chi-square	*P*
Sex				23.289	<0.001
Men	864	661	76.5		
Women	1596	1347	84.4		
Age group (years)				33.560	<0.001
18-29	520	459	88.3		
30-39	920	761	82.7		
40-49	471	377	80.0		
≥50	549	411	74.9		
Occupation				46.251	<0.001
Nurse	753	667	88.6		
Doctor	560	440	78.6		
Medical technician	244	209	85.7		
Support staff	683	528	77.3		
Administrator	220	164	74.5		
Total	2460	2008	81.6		

### 3.2 Description of participants in the questionnaire survey

We collected 1,742 valid questionnaires, resulting in a response rate of 70.8%. A total of 520 (29.9%) men and 1,222 (70.1%) women were included, with an average age of 37.6 ± 10.3 years. Nurses (35.9%), doctors (25.8%), support staff (20.4%), medical technicians (12.8%), and administrators (5.1%) participated. Among the 1,742 participants, 1,463 (84.0%) tested positive for SARS-CoV-2; 33.4% were RT-PCR positive, and 66.6% were RAT positive.

### 3.3 Clinical characteristics of SARS-CoV-2 infection

Among the 1,463 SARS-CoV-2 positive-HCWs, 1,392 (95.1%) had COVID-19-related symptoms, and the remaining 71 (4.9%) were asymptomatic. Among the 1392 symptomatic infections, fever (88.4%) was the most common symptom, followed by cough (84.5%), fatigue (75.9%), sore throat (63.6%) and myalgia (55.0%). For the duration of symptoms, cough lasted the longest (15.2 ± 10.7 days), followed by fatigue (11.7 ± 10.3 days) and anosmia (9.4 ± 9.3 days). Among patients with symptomatic infections, 10.6% had outpatient visits, 0.6% required hospitalisation, 1.1% received oxygen therapy, and 4.4% developed pneumonia (Table 2).

**Table 2:** Clinical characteristics of symptomatic SARS-CoV-2 infections, N = 1392.

Clinical characteristics		Number	Percentage (%)	Duration (days)		
				Minimum	Maximum	Mean ± SD
Symptom	Fever	1231	88.4	1.0	45.0	3.0 ± 2.1
	Highest temperature (°C)	1231	39.1 ± 0.7^a^	–	–	–
	Sore throat	886	63.6	0.5	50.0	5.0 ± 4.1
	Cough	1176	84.5	0.5	77.0	15.2 ± 10.7
	Nasal congestion and runny nose	737	52.9	0.5	50.0	6.4 ± 5.7
	Fatigue	1056	75.9	0.5	60.0	11.7 ± 10.3
	Myalgia	766	55.0	0.5	50.0	4.7 ± 5.1
	Diarrhea	229	16.5	0.5	30.0	2.9 ± 4.1
	Ageusia	420	30.2	0.5	60.0	8.5 ± 8.3
	Anosmia	337	24.2	0.5	60.0	9.4 ± 9.3
	Chest distress	331	23.8	0.5	50.0	8.7 ± 8.8
	Short of breath or difficult breathing	232	16.7	0.5	50.0	7.4 ± 9.0
	Conjunctivitis	70	5.0	0.5	45.0	3.8 ± 8.0
Treatment	Outpatient visit	148	10.6	–	–	–
	Hospitalization	9	0.6	–	–	–
	Oxygen therapy	16	1.1	–	–	–
	Pneumonia	61	4.4	–	–	–

a Mean ± SD.

### 3.4 Source of SARS-CoV-2 infection

The source of infection was categorised as inside or outside the hospital. Inside the hospital was defined as having contact with positive patients or colleagues or contaminated surroundings in the workplace in the past 14 days before testing positive for SARS-CoV-2 in the absence of exposure to positive family members or friends outside the hospital. Outside the hospital was defined as the opposite behaviours. Among the 1,463 infected HCWs, 776 (53.0%) had gotten infected outside the hospital and 644 (44.0%) in the hospital, of whom 333 (51.7%) had exposure to positive colleagues, 214 (33.2%) positive patients and 97 (15.1%) contaminated surroundings; 43 (2.9%) could not identify the source of the infection.

### 3.5 Risk factors for SARS-CoV-2 infection in HCWs

Table 3 shows the distribution of risk factors between infected and uninfected HCWs, as well as the univariate and multivariate logistic regression models for the risk of SARS-CoV-2 infection. As revealed by multivariate analysis, older HCWs were less likely to be infected (aOR 0.97, 95% CI 0.94 to 0.99, *P* = 0.010, for each year increase in age). Compared with HCWs who were working in the non-clinic area, those working in the emergency department (aOR 3.77, 95% CI 1.69 to 8.38, *P* = 0.001) and in the medical technology area (aOR 2.47, 95% CI 1.10 to 5.51, *P* = 0.028) were associated with higher odds of infection. HCWs who had previous infection with SARS-CoV-2 6 months prior were less likely to be infected than those who did not (aOR 0.01, 95% CI 0 to 0.07, *P* < 0.001). Those who had received four vaccine doses were less likely to be infected than those who were unvaccinated (aOR 0.40, 95% CI 0.17 to 0.97, *P* = 0.042).

**Table 3:** Risk factors of SARS-CoV-2 infection in HCWs by univariate and multivariate logistic regression analysis.

Category	Factor	SARS-CoV-2 infected N = 1463 n (%)	SARS-CoV-2 uninfected N = 279 n (%)	Univariate analysis		Multivariate analysis	
				OR (95%CI)	*P*	adjusted OR (95%CI)	*P*
Demographics	Sex						
	Men	421 (28.8)	99 (35.5)	Reference		Reference	
	Women	1042 (71.2)	180 (64.5)	1.36 (1.04–1.78)	0.025	1.27 (0.92–1.75)	0.154
	Age (years, linear term)	37.3 ± 10.1^a^	39.4 ± 11.2^a^	0.98 (0.97–0.99)	0.002	0.97 (0.94–0.99)	0.010
	Occupation						
	Administrator	69 (4.7)	20 (7.2)	Reference		Reference	
	Nurse	544 (37.2)	81 (29.0)	1.95 (1.12–3.37)	0.018	0.88 (0.39–1.99)	0.757
	Doctor	389 (26.6)	61 (21.9)	1.85 (1.05–3.26)	0.033	1.58 (0.72–3.45)	0.254
	Medical technician	183 (12.5)	40 (14.3)	1.33 (0.72–2.43)	0.360	0.68 (0.30–1.53)	0.354
	Support staff	278 (19.0)	77 (27.6)	1.05 (0.60–1.83)	0.873	1.04 (0.41–2.64)	0.939
	Education						
	Below undergraduate	433 (29.6)	101 (36.2)	Reference		Reference	
	Undergraduate	633 (43.3)	94 (33.7)	1.57 (1.16–2.13)	0.004	0.96 (0.58–1.58)	0.869
	Master or above	397 (27.1)	84 (30.1)	1.10 (0.80–1.52)	0.550	0.64 (0.34–1.20)	0.165
Work characteristic	Work category^b^						
	Frontline work	1190 (81.3)	205 (73.5)	Reference		Reference	
	Second-line work	273 (18.7)	74 (26.5)	0.64 (0.47–0.85)	0.003	1.04 (0.66–1.64)	0.857
	Work site						
	Non-clinic area	104 (7.1)	40 (14.3)	Reference		Reference	
	Outpatient setting	259 (17.7)	44 (15.8)	2.26 (1.39–3.68)	0.001	1.65 (0.82–3.32)	0.161
	Emergency department	210 (14.4)	16 (5.7)	5.05 (2.70–9.44)	<0.001	3.77 (1.69–8.38)	0.001
	Medical examination area	105 (7.2)	16 (5.7)	2.52 (1.33–4.79)	0.005	2.47 (1.10–5.51)	0.028
	Inpatient ward	528 (36.1)	95 (34.1)	2.14 (1.40–3.27)	<0.001	1.37 (0.71–2.65)	0.347
	Fever clinic and isolation ward	77 (5.3)	27 (9.7)	1.10 (0.62–1.94)	0.751	1.06 (0.49–2.29)	0.887
	Operating room	98 (6.7)	22 (7.9)	1.71 (0.95–3.09)	0.073	1.12 (0.51–2.44)	0.773
	Support department	82 (5.6)	19 (6.8)	1.66 (0.90–3.08)	0.108	1.64 (0.81–3.32)	0.172
Previous medical history	Underlying disease^c^						
	No	1363 (93.2)	256 (91.8)	Reference		Reference	
	Yes	100 (6.8)	23 (8.2)	0.82 (0.51–1.31)	0.401	0.73 (0.44–1.23)	0.241
	Previous infection with SARS-CoV-2^d^						
	No	1461 (99.9)	264 (94.6)	Reference		Reference	
	Yes	2 (0.1)	15 (5.4)	0.02 (0.01–0.11)	<0.001	0.01 (0.00–0.07)	<0.001
Vaccination status	Doses of vaccine (by 31 Jan 2023)						
	0 dose	44 (3.0)	7 (2.5)	Reference		Reference	
	1 dose	23 (1.6)	8 (2.9)	0.46 (0.15–1.42)	0.176	0.52 (0.16–1.70)	0.279
	2 doses	179 (12.2)	26 (9.3)	1.10 (0.45–2.69)	0.842	1.34 (0.53–3.40)	0.536
	3 doses	1076 (73.5)	165 (59.1)	1.04 (0.46–2.34)	0.929	1.36 (0.59–3.17)	0.471
	4 doses	141 (9.6)	73 (26.2)	0.31 (0.13–0.72)	0.006	0.40 (0.17–0.97)	0.042

a Mean ± SD.

b Frontline work referred to work with patient-facing tasks; second-line work referred to work without patient-facing tasks.

c Underlying diseases included hypertension, diabetes, hyperlipidemia, heart disease, cerebrovascular disease, chronic lung disease, chronic kidney disease, malignant tumor, immunosuppression/immunodeficiency, etc.

d All previous infections with SARS-CoV-2 occurred in the first half of 2022 (February to May), when the Omicron variant predominated, over 6 months before the beginning of the study.

### 3.6 Risk factors for SARS-CoV-2 infection in frontline HCWs

We explored the effects of patient contact and personal protection on the risk of SARS-CoV-2 infection among frontline HCWs. In the multivariate logistic regression analysis, after controlling for all the variables shown in Table 3 except for ‘work category’, we found that HCWs who had oral-nasal exposure to coworkers, such as taking off their mask during breaks, eating, or talking in the presence of colleagues, were more likely to be infected compared with those without the above situations (aOR 1.74, 95%CI 1.21 to 2.51, *P* = 0.003) (Table 4).

**Table 4:** Risk factors of SARS-CoV-2 infection in frontline HCWs by univariate and multivariate logistic regression analysis.

Category	Factor	SARS-CoV-2 infected N = 1190 n (%)	SARS-CoV-2 uninfected N = 205 n (%)	Univariate analysis		Multivariate analysis^a^	
				OR (95%CI)	*P*	adjusted OR (95%CI)	*P*
Patient contact	Total time of patient contact per day (hours)						
	<4	174 (14.6)	36 (17.6)	Reference		Reference	
	4–8	382 (32.1)	64 (31.2)	1.23 (0.79–1.93)	0.354	1.02 (0.62–1.68)	0.946
	≥8	634 (53.3)	105 (51.2)	1.25 (0.83–1.89)	0.292	0.91 (0.56–1.49)	0.713
	Contact time per patient (minutes)						
	<5	244 (20.5)	53 (25.9)	Reference		Reference	
	5–15	529 (44.5)	69 (33.7)	1.67 (1.13–2.46)	0.010	1.55 (0.98–2.43)	0.059
	16–30	123 (10.3)	27 (13.2)	0.99 (0.59–1.65)	0.968	1.13 (0.62–2.07)	0.685
	≥30	294 (24.7)	56 (27.3)	1.14 (0.76–1.72)	0.532	1.18 (0.72–1.95)	0.512
	Distance from patient						
	Touched patient	931 (78.2)	152 (74.1)	Reference		Reference	
	1 metre away	177 (14.9)	40 (19.5)	0.72 (0.49–1.06)	0.097	0.79 (0.51–1.22)	0.283
	2 metre away	17 (1.4)	4 (2.0)	0.69 (0.23–2.09)	0.516	0.92 (0.25–3.36)	0.900
	No contact but in the same room	65 (5.5)	9 (4.4)	1.18 (0.58–2.42)	0.653	2.14 (0.89–5.17)	0.090
Personal protection	PPE						
	None	454 (38.2)	87 (42.4)	Reference		Reference	
	Isolation gown	579 (48.7)	77 (37.6)	1.44 (1.04–2.01)	0.030	1.31 (0.90–1.93)	0.161
	Protective clothing	157 (13.2)	41 (20.0)	0.73 (0.49–1.11)	0.142	0.99 (0.61–1.61)	0.969
	Oral-nasal exposure to coworkers^b^						
	No	285 (23.9)	69 (33.7)	Reference		Reference	
	Yes	905 (76.1)	136 (66.3)	1.61 (1.17–2.22)	0.003	1.74 (1.21–2.51)	0.003
	Duration of oral-nasal exposure per day (hours)						
	<1	971 (81.6)	171 (83.4)	Reference		Reference	
	1–2	145 (12.2)	26 (12.7)	0.98 (0.63–1.54)	0.937	0.78 (0.48–1.27)	0.323
	≥2	74 (6.2)	8 (3.9)	1.63 (0.77–3.44)	0.201	1.54 (0.69–3.45)	0.294

a Multivariate logistic regression analysis after controlling for all the variables shown in Table 3 except for ‘work category’ (see Table 4).

b Oral-nasal exposure to coworkers included the following situations: taking off the mask during breaks, eating, or talking in the presence of colleagues.

### 3.7 COVID-19 vaccine effectiveness

After excluding HCWs with previous SARS-CoV-2, 1,725 were included in the analysis of vaccine effectiveness. By 1 December 2022, at the beginning of the study period, 80.3% of the HCWs had received three doses of the COVID-19 vaccine, 14.9% had received two doses, 1.8% had received one dose, and 3.0% were unvaccinated. In December 2022, an emergency booster vaccination of the adenovirus vector vaccine, the ‘Cansino Ad5-nCoV-S COVID-19 vaccine’, was administered to HCWs. By 31 December 2022, 5.9% of the HCWs had received four doses, 75.7% had received three doses, 13.7% had received two doses, 1.8% had received one dose, and 3.0% were unvaccinated. No one was vaccinated in January 2023. The various vaccination statuses (classified by the number of vaccine doses and time since the last dose), vaccination course, and vaccine type, are shown in Table 5.

**Table 5:** Vaccination statuses classified by the number of vaccine doses, time since the last dose, vaccination course, and vaccine type, n (%).

	Unvaccinated	Partial vaccination^c^	Full vaccination^d^		Homologous booster vaccination^e^		Heterologous booster vaccination^f^		Total
		1 dose of inactivated^a^	2 doses of inactivated	1 dose of Cansino^b^	3 doses of inactivated	2 doses of Cansino	3 doses of inactivated + 1 dose of Cansino	2 doses of inactivated + 1 dose of Cansino	
0 dose	51 (2.96)								51 (2.96)
1 dose <6 months		4 (0.23)		3 (0.17)					7 (0.41)
1 dose ≥6 months		20 (1.16)		4 (0.23)					24 (1.39)
2 doses <6 months			6 (0.35)			2 (0.12)			8 (0.46)
2 doses ≥6 months			228 (13.22)			1 (0.06)			229 (13.28)
3 doses <6 months					38 (2.20)			4 (0.23)	42 (2.43)
3 doses ≥6 months					1243 (72.06)				1243 (72.06)
3 doses during the epidemic					3 (0.17)			17 (0.99)	20 (1.16)
4 doses during the epidemic							101 (5.86)		101 (5.86)

a Inactivated vaccines included Sinopharm/BIBP COVID-19, Sinopharm/WIBP COVID-19 and Sinovac-CoronaVac.

b Cansino was the adenovirus vector vaccine called ‘Cansino Ad5-nCoV-S COVID-19 vaccine’.

c Partial vaccination was receiving only one dose of inactivated vaccine.

d Full vaccination was receiving two doses of inactivated vaccines or one dose of Cansino.

e Homologous booster vaccination was receiving three doses of inactivated vaccines or two doses of Cansino.

f Heterologous booster vaccination was receiving two or three doses of inactivated vaccines plus one dose of Cansino.

The incidence rates of primary SARS-CoV-2 infection stratified by vaccination status are shown in Table 6. The incidence rate in HCWs who received the fourth dose of the vaccine during the epidemic was significantly lower than in those who received 0, 1, 2, or 3 doses. HCWs who received heterologous boosters had a lower incidence rate than those who received homologous boosters (32.8% vs 88.9%). In the univariate Cox regression analysis, compared with HCWs who were unvaccinated, those who received the fourth dose during the epidemic were less likely to acquire infection (HR 0.25, 95% CI 0.15 to 0.40, *P* < 0.001). Protection from the heterologous booster of two or three doses of inactivated vaccines plus one dose of adenovirus vector vaccine was higher than the homologous booster of three doses of inactivated vaccines (HR 0.24, 95% CI 0.18 to 0.33, *P* < 0.001).

**Table 6:** Incidence rates of primary SARS-CoV-2 infection by vaccination status and univariate Cox proportional regression model for the risk of primary infection from 1 December 2022 to 31 January 2023.

Vaccination status	Total number of HCWs	Events of SARS-CoV-2 infection	Incidence rate (%)	Univariate analysis	
				HR (95%CI)	*P*
Vaccine doses by time since the last dose					
0 dose	51	44	86.3	Reference	
1 dose <6 months	7	3	42.9	0.38 (0.12–1.22)	0.105
1 dose ≥6 months	24	20	83.3	1.28 (0.75–2.17)	0.361
2 doses <6 months	8	7	87.5	1.43 (0.65–3.18)	0.377
2 doses ≥6 months	229	206	90.0	1.31 (0.95–1.82)	0.103
3 doses <6 months	42	36	85.7	1.05 (0.67–1.63)	0.834
3 doses ≥6 months	1243	1106	89.0	1.31 (0.97–1.78)	0.076
3 doses during the epidemic	20	11	55.0	0.75 (0.39–1.46)	0.403
4 doses during the epidemic	101	28	27.7	0.25 (0.15–0.40)	<0.001
Type of booster					
Homologous booster^a^	1284	1141	88.9	Reference	
Heterologous booster^b^	122	40	32.8	0.24 (0.18–0.33)	<0.001

a Homologous booster was three doses of inactivated vaccines, excluding two doses of adenovirus vector vaccines (Cansino).

b Heterologous booster was two or three doses of inactivated vaccines plus one dose of adenovirus vector vaccine (Cansino).

Table 7 shows the results of the multivariate Cox proportional regression analysis controlling for the factors involved in the logistic regression model. Compared with HCWs who were unvaccinated, those who received the fourth dose of vaccine (the emergency booster during the epidemic) displayed a strong protective effect (aHR 0.25, 95% CI 0.15 to 0.40, *P* < 0.001), resulting in an adjusted-VE of 75.1% (95% CI 84.6%–59.5%). Factors in the logistic regression model, including age and work site, remained significant in the Cox regression model.

**Table 7:** Multivariate Cox proportional regression model for the risk of primary SARS-CoV-2 infection from 1 December 2022 to 31 January 2023.

Category	Factor	Univariate analysis		Multivariate analysis	
		HR (95%CI)	*P*	adjusted HR (95%CI)	*P*
Demographics	Sex				
	Men	Reference		Reference	
	Women	1.10 (0.98–1.23)	0.094	1.10 (0.97–1.26)	0.135
	Age (years, linear term)	1.00 (0.99–1.00)	0.214	0.99 (0.98–1.00)	0.011
	Occupation				
	Administrator	Reference		Reference	
	Nurse	1.18 (0.92–1.52)	0.193	0.95 (0.70–1.30)	0.760
	Doctor	1.23 (0.95–1.59)	0.109	1.13 (0.83–1.53)	0.440
	Medical technician	0.97 (0.74–1.28)	0.832	0.74 (0.54–1.02)	0.068
	Support staff	1.14 (0.88–1.48)	0.329	1.15 (0.80–1.64)	0.459
	Education				
	Below undergraduate	Reference		Reference	
	Undergraduate	0.96 (0.85–1.08)	0.473	0.85 (0.71–1.02)	0.076
	Master or above	0.94 (0.82–1.07)	0.336	0.84 (0.66–1.06)	0.142
Work characteristic	Work category				
	Frontline work	Reference		Reference	
	Second-line work	0.94 (0.82–1.07)	0.325	1.11 (0.93–1.34)	0.252
	Work site				
	Non-clinic area	Reference		Reference	
	Outpatient setting	1.31 (1.04–1.65)	0.020	1.32 (0.99–1.77)	0.063
	Emergency department	1.69 (1.33–2.14)	0.000	1.67 (1.24–2.25)	0.001
	Medical technology area	1.35 (1.03–1.77)	0.031	1.60 (1.16–2.21)	0.004
	Inpatient ward	1.23 (1.00–1.52)	0.050	1.17 (0.88–1.55)	0.291
	Fever clinic and isolation ward	0.96 (0.71–1.28)	0.766	1.19 (0.84–1.68)	0.330
	Operating room	1.05 (0.80–1.39)	0.711	0.99 (0.71–1.39)	0.973
	Support department	1.36 (1.02–1.81)	0.039	1.32 (0.97–1.81)	0.077
Underlying disease					
	No	Reference		Reference	
	Yes	0.86 (0.70–1.06)	0.161	0.95 (0.77–1.17)	0.600
Vaccination status	Vaccine doses by time since the last dose				
	0 dose	Reference		Reference	
	1 dose <6 months	0.38 (0.12–1.22)	0.105	0.41 (0.13–1.34)	0.140
	1 dose ≥6 months	1.28 (0.75–2.17)	0.361	1.18 (0.69–2.02)	0.549
	2 doses <6 months	1.43 (0.65–3.18)	0.377	1.47 (0.66–3.28)	0.349
	2 doses ≥6 months	1.31 (0.95–1.82)	0.103	1.29 (0.92–1.80)	0.133
	3 doses <6 months	1.05 (0.67–1.63)	0.834	1.00 (0.64–1.56)	0.989
	3 doses ≥6 months	1.31 (0.97–1.78)	0.076	1.30 (0.95–1.77)	0.097
	3 doses during the epidemic	0.75 (0.39–1.46)	0.403	0.72 (0.37–1.40)	0.328
	4 doses during the epidemic	0.25 (0.15–0.40)	<0.001	0.25 (0.15–0.40)	<0.001

## 4 Discussion

During the 28-day COVID-19 outbreak observed in our study, 81.6% of the HCWs were infected, as shown in the surveillance data. Based on national data, the epidemic curve and infection rate were similar to those of the general population from the national data. According to a recent study by the Chinese Center for Disease Control and Prevention through an online survey performed in 31 provinces, the self-reported infection rate of the national population was estimated to be 82.4% by 7 February 2023 [11]. However, the infection rate in our study was higher than that reported in other countries. Based on a systematic review, the prevalence of COVID-19 in HCWs was reported as 51.7% (95% CI 34.7 to 68.2) [14]. This discrepancy may depend on the variance in prevention and control policy, surveillance and testing strategy, epidemic period, predominant variant strain, population immunity, vaccination status, and infection control behaviour influenced by HCWs' perception or training across different countries and regions. This was the first Omicron wave encountered by HCWs since the Chinese government relaxed the prevention and control strategies in early December 2022, and before which strict public health interventions had been implemented for a long time. During the Omicron wave in our study, the mandatory daily testing for SARS-CoV-2 enforced on HCWs enabled us to detect all asymptomatic infections, acquired far outside the hospital. However, due to the explosive increase in infected patients in the community, the hospital's medical treatment capacity and workload of HCWs were vastly overstretched, which hindered HCW's compliance with IPC measures. Moreover, despite the high vaccination coverage in HCWs, post-vaccination breakthrough infection caused by the immune escape of Omicron was inevitable, and protection from vaccination waned over time as the last dose was over 6 months in most HCWs.

With regard to clinical characteristics, the vast majority (95.1%) of the infected HCWs were symptomatic, with fever, cough, fatigue, sore throat and myalgia being the five most common symptoms, which is consistent with the results from Wuhan [15]. Most infected HCWs had mild symptoms; only 0.6% required hospitalisation, and 1.1% received oxygen therapy. Many studies have reported that older patients with underlying conditions have much higher risk of developing severe illness from COVID-19 than the general population [9,16]. In our study population of HCWs, the majority were young, only 7.1% had underlying conditions, and infections in HCWs were more easily identified early, with better access to treatment than in the general population. All these factors probably resulted in fewer patients developing severe disease.

Younger age was associated with an increased risk of infection in our study, consistent with the findings of previous studies [[17], [18], [19]]. Three possible reasons can explain this result: First, younger HCWs had less risk perception and inadequate adherence to IPC measures, such as proper use of personal protective equipment (PPE) and social distancing. Second, younger HCWs spent a greater proportion of working time in face-to-face patient care compared with elder ones. Third, the sources of infection among younger HCWs were not only from the workplace but also from the community because of their more frequent social activities outside the hospital, increasing their risk of infection. The latter two explanations were supported by three cohort studies on the same large population of HCWs, followed up over time before and during the Omicron period [[20], [21], [22]].

As for another core demographic factor of sex, although the infection rate was higher in women HCWs than their men counterparts, there was no significant association between sex and SARS-CoV-2 infection in the multivariate analysis. The impact of sex on COVID-19 acquisition reported from existing literatures varied across different periods of epidemic [18,19,23]. Recent studies reported an inverse infection risk with regard to sex, with higher risk in men before the Omicron period and lower risk in men during the Omicron epidemic [21,22]. During the pre-Omicron period, when humoral immunity was still building up, men were more likely to be infected and develop severe COVID-19. By contrast, during the Omicron wave, the evasion capacity probably enabled Omicron to overcome humoral immunity. This might explain the higher risk of infection in women, who accounted for the majority of infections and were more likely to be assigned to high-risk job tasks involving patient contacts especially as nurses and nurse aids [20].

Nurses and medical assistants have been frequently reported as professionals at a higher risk of occupational COVID-19 in several studies [6,17,19,[21], [22], [23], [24], [25], [26]]. Although we observed higher infection rates among nurses and medical technicians, the impact of occupation was not confirmed at multivariate analysis. Moreover, we did not find a higher risk of infection among frontline HCWs compared with second-line HCWs. A greater proportion of nurses worked on the frontline with more patient contacts than those in other occupational categories. Our results implied that the occupational category had no effect on the infection risk among HCWs. In Italy during the Omicron transmission period, in highly vaccinated populations such as HCWs, a massive number of SARS-CoV-2 infections were predominantly acquired outside the hospital, where mandatory risk-reduction measures (face mask, social distancing, etc.) were less stringently enforced [21]. Therefore, HCWs face a dual risk of infection, not only from the workplace but also principally from the community. Although frontline HCWs had a higher frequency and intensity of exposure to infected patients than second-line HCWs, they had greater risk perception, proper usage of PPE, and better adherence to IPC measures to protect themselves against infections outside the hospital. Both were exposed to a high risk of infection from the community, but it is possible that second-line HCWs had a higher risk of infection due to less stringent adherence to IPC.

Although not associated with occupation, the risk of infection increased significantly in some workplaces, such as emergency departments and medical examination areas, similar to other studies on HCWs in India, Italy, and Spain [15,18,22,25]. The possible explanation for the high risk of infection in emergency departments might be the emergency context and a high number of casualties, which make it more difficult to strictly observe health protection measures [21]. The medical examination area was not considered high-risk area for infection, which probably resulted in low-risk perception and inadequate adherence to PPE usage and IPC measures among HCWs working in these zones. In contrast, in the fever clinic and isolation ward, known as the most hazardous area for high levels of exposure to the virus, the risk of infection was the lowest and closest to that in the non-clinic area. Three possible explanations can provide insights into this finding: First, HCWs who worked in these zones were required to wear isolation gowns with sufficient PPE and received pre-post training and examination on wearing and removing PPE, and they were also under intensive supervision of daily on-duty behaviours. Second, they had a stronger awareness of infection risk and better compliance with stringent IPC measures. Third, complete vaccination was one of the necessary inclusion criteria for HCWs who worked in these zones. These findings emphasise a greater impact of workplace than job tasks on infection risk and the important role of strict observation of IPC measures in protecting HCWs against infection during the pandemic [20].

The most common source of SARS-CoV-2 infection in HCWs was contact with positive family members or friends outside the workplace during the Omicron wave, which was in accordance with several studies in the open literature. Studies from Italy and USA found that most HCWs acquired SARS-CoV-2 infections outside the workplace after the second COVID-19 wave, but no occupational factors were found to be associated with an increased risk of infection [21,24,27]. It was envisaged that during the epidemic period, risk-reduction rules were enforced and strictly observed among HCWs in the hospital; hence, infections should have been acquired outside the workplace, where mask wearing, hand-washing, and social distancing were less stringently enforced. Furthermore, there is evidence that after the early stage of the pandemic, the HCWs’ compliance with PPE use improved, hence the occupational infection rates gradually declined during the subsequent waves, despite the increased risk of SARS-CoV-2 contagion over time [21].

In addition, it should be noted that a greater number of infections in the workplace were from contact with positive colleagues than with positive patients. A study from Switzerland showed that caring for isolated patients did not increase the risk of infection, but working shifts with pre-symptomatic healthcare colleagues did [28]. The primary reason for this finding might be the occurrence of oral-nasal exposure to coworkers on duty, such as taking off the mask during eating, breaks, or talking in the presence of colleagues because we also found a higher risk of infection among HCWs with the above oral-nasal exposures than among those without. When dealing with colleagues in the workplace, HCWs are more likely to inadvertently relax their attention to risk-reduction behaviour, as reported elsewhere [21]. Therefore, it is crucial to emphasise adherence to IPC measures, such as universal masking and social distancing, to prevent the spread of nosocomial infections among colleagues during the pandemic.

In our study, a strong protective factor associated with a lower risk of COVID-19 was previous infection with SARS-CoV-2. Several studies have documented the protective effect of past infection in reducing the risk of re-infection, but the protection efficacy declined due to immunity wanes over time and emergence of Omicron variants [5]. In our study, all previous infections occurred in the first half of 2022 (February to May), when the Omicron variant predominated, with a timeline of 7–10 months before the beginning of the Omicron outbreak on 1 December 2022. It is reasonable that the protection acquired from previous infection affected re-infection prevention. In contrast, all HCWs with a previous infection received two or three doses of the COVID-19 vaccine, implying that hybrid humoral immunity by vaccination combined with natural infection provided higher protection than vaccination alone. Further studies with longer observations and larger sample sizes are needed to estimate the protection from past infection against re-infection by variants and time since infection in the future.

In our study, the high vaccination coverage and mandatory daily testing schedule among HCWs allowed us to evaluate the vaccine effectiveness against SARS-CoV-2 infection under various vaccination statuses. Although the COVID-19 vaccination uptake of at least one dose was as high as 96.8% before the Omicron pandemic, the protection from vaccination against SARS-CoV-2 infection was not optimal, as the risk of infection in HCWs who received two or three doses of the vaccine was not lower than that in unvaccinated HCWs. Immune escape is observed in the Omicron variant, which features a higher risk of re-infection than the previous variants, regardless of preexisting immunity, which led to breakthrough infections after vaccination in many countries worldwide [21]. Additionally, the time elapse after vaccination and the decline in antibody levels also attribute in breakthrough infections [20]. Therefore, the most likely explanation was that 85.5% of the HCWs received the last dose of the vaccine over 6 months before infection, most of whom participated in the vaccination program centrally organised by SGH before November 2021 in the pre-Omicron period, with a time interval of over 1 year. However, another study on the general population in China during the same epidemic period reported different results. The effectiveness of booster vaccinations against self-reported Omicron infection was 49% within 3 months and 38% between 3 and 6 months after vaccination [11]. This discrepancy can be explained by the different testing schedules of HCWs and the general population. During the Omicron transmission period, infections were almost mild or asymptomatic; hence, a mandatory daily testing screening schedule in HCWs could detect all asymptomatic infections. In contrast, in the latter study in the general population, the testing was voluntary only in case of developing symptoms related to COVID-19; therefore, asymptomatic infections were inevitably missed. The vaccine effectiveness in the present study probably reflects protection against asymptomatic COVID-19. These findings are supported by two Italian studies of HCWs and university staff [22,29].

Encouragingly, we discovered a strong protective effect of the emergency booster vaccination (the fourth dose with a heterologous booster of the adenovirus vector vaccine following three doses of inactivated vaccines) administered during the Omicron pandemic against primary SARS-CoV-2 infection, with an estimated adjusted VE of 75.2%. To fight against the problem of immune escape associated with the Omicron variant, many countries began to implement booster vaccination to restore protection against infection by increasing the neutralising activity and T-cell response [3]. Many studies have demonstrated that heterologous booster vaccination produces a more robust immune response than homologous booster vaccination. According to a recent systematic review and meta-analysis, in addition to completing two doses of inactivated vaccines, compared with homologous boosters, heterologous boosters with mRNA vaccine and non-replicating vector vaccine showed higher effectiveness against COVID-19 with VE of 89.19% and 87.00%, respectively [30].

To date, this is the first exploration in China to evaluate the effectiveness of emergency booster vaccination during the Omicron epidemic among HCWs in real life, with stronger validity after controlling for several confounding factors. However, it is regrettable that the emergency booster vaccination was launched too late at the peak of the Omicron wave. Many HCWs lost the chance to receive the booster because they had contracted infections. Therefore, it is reasonable to presume a stronger protective effect against infection if booster vaccination among HCWs was initiated before the Omicron epidemic. It is also important to inform public health policies to recommend vaccination to vulnerable individuals at risk of developing severe COVID-19 before future pandemic waves.

Our study had several limitations. First, recall bias was inevitable because the questionnaire was conducted after the outbreak, and exposure among HCWs was self-reported. Second, some cases were confirmed by RAT with lower sensitivity and quality control than the gold standard of the PCR test, which might have led to questions about reliability and validity. Third, the generalisability of the findings was limited by the single-centre study conducted in a tertiary hospital; therefore, further multi-centre studies are required. Lastly, in the VE analysis, an insufficient sample size for some categories of vaccination status (one dose <6 months, two doses <6 months) resulted in reduced statistical power with wide CIs.

## 5 Conclusions

This case-control study reported the epidemiological characteristics of the Omicron pandemic among HCWs in Shanghai and identified key demographic and occupational risk factors associated with an increased risk of SARS-CoV-2 infection. We also found a protective effect of emergency heterologous booster vaccination against primary SARS-CoV-2 infection. This should inform public health policies to aim at protecting HCWs in response to future pandemic waves by means of individualised risk assessments, adopting risk-differentiated IPC measures, intensive training and supervision of infection control behaviours. Particular emphasis should be placed on the importance of personal protection among coworkers and recommending vaccination to vulnerable individuals at risk of developing severe COVID-19 before the arrival of another Omicron wave.

## Ethics statement

This study was reviewed and approved by the Institutional Review Board of SGH, with the approval number: 2023-156. Implied informed consent was obtained from all participants on submission of questionnaire in the study.

## Funding statement

This study was supported by Science and Technology Commission of Songjiang District, Shanghai Municipality (grant numbers 21SJKJGG94, 21SJKJGG148), and 10.13039/501100008750Shanghai Hospital Development Center (grant number SHDC22022211).

## Data availability statement

Data associated with this study has been deposited at Mendeley Data (https://data.mendeley.com/) with https://doi.org/10.17632/jxd96skyvv.2 [31].

## CRediT authorship contribution statement

**Dan Wang:** Writing – review & editing, Writing – original draft, Software, Project administration, Methodology, Investigation, Formal analysis, Data curation, Conceptualization. **Dan Zhu:** Investigation, Data curation. **Min Xia:** Investigation, Data curation. **Xiaoying Wang:** Investigation, Data curation. **Ni Zou:** Validation, Supervision, Resources, Project administration, Funding acquisition, Conceptualization.

## Declaration of competing interest

The authors declare that they have no known competing financial interests or personal relationships that could have appeared to influence the work reported in this paper.
