# Supplementary material for: Epidemiology, risk factors, and vaccine effectiveness for SARS-CoV-2 infection among healthcare workers during the omicron pandemic in Shanghai, China
Source: Heliyon. 2024 May 29;10(11):e32182. doi: 10.1016/j.heliyon.2024.e32182 (PMC11214455; doi:10.1016/j.heliyon.2024.e32182)
Supplement: Multimedia component 1 [file mmc1.docx]

**Questionnaire survey of COVID-19 infection in healthcare workers**

In order to understand the epidemiology and risk factors of COVID-19 infection among healthcare workers during the Omicron pandemic between December 2022 and January 2023, a questionnaire survey of COVID-19 infection was conducted among healthcare workers who worked in Shanghai General Hospital. The questionnaire survey is completely anonymous and voluntary, and for hospital staff only (including nurses, doctors, medical technicians, administrators and support staff). No matter whether you have been infected with COVID-19, we hope you can actively participate in this survey and provide us with valuable data for epidemiological research and decision making of infection prevention and control. Thank you for your support to our study!

**Ⅰ Basic information**

1. Sex

A. Male

B. Female

2. Date of birth: yyyy-mm-dd

3. Department: ________________

4. Occupation:

A. Doctor

B. Nurse

C. Medical technician

D. Administrator

E. Support staff (cleaners, nursing worker, security guards, delivery worker, maintenance staff, catering staff)

F. Others________________

5. Education:

A. Below undergraduate

B. Undergraduate

C. Master or above

6. Work site: (multiple choices)

A. Outpatient

B. Emergency

C. Medical technology area

D. Inpatient ward

E. ICU

F. Maternity ward

G. Newborn room

H. Laminar flow ward

I. Hemodialysis room

J. Fever clinic/isolation ward

K. Central sterile supply department (CSSD)

L. Operating room

M. Administrative building

N. Scientific research building/laboratory building

O. Dining hall

P. Basement (logistic support)

Q. Others________________

**Ⅱ Infection status**

1. Have you ever been confirmed with COVID-19 infection since December 2022? (nucleic acid positive or antigen positive)

A. Yes

B. No (skip to 4)

2. Diagnosis method:

A. Nucleic acid positive

B. Antigen positive

3. Diagnosis date: yyyy-mm-dd (skip to 5)

4. Did you have a suspected COVID-19 infection? (had COVID-19 related symptoms but not tested for nucleic acid or antigen)

A. Yes

B. No (skip to Ⅲ)

5. Did you have COVID-19 related symptoms?

A. Yes

B. No (skip to 14)

6. Date of initial symptom onset: yyyy-mm-dd

7. Did you have fever?

A. Yes

B. No (skip to 9)

8. Highest temperature of fever: ____℃, duration of fever: ____days

9. Did you have the following symptoms? (multiple choices)

A. Sore throat (lasted for ____ days)

B. Cough (lasted for ____ days)

C. Nasal congestion and runny nose (lasted for ____ days)

D. Fatigue (lasted for ____ days)

E. Myalgia (lasted for ____ days)

F. Diarrhea (lasted for ____ days)

G. Ageusia (lasted for ____ days)

H. Anosmia (lasted for ____ days)

I. Chest distress (lasted for ____ days)

J. Short of breath/difficult breathing (lasted for ____ days)

K. Conjunctivitis (lasted for ____ days)

L. Others_______________

10. Did you have outpatient visit?

A. Yes

B. No

11. Were you hospitalized?

A. Yes

B. No

12. Did you receive oxygen treatment?

A. Yes

B. No

13. Did you have pneumonia?

A. Yes

B. No

C. No clear

14. Negative conversion:

A. Negative conversion of nucleic acid

B. Negative conversion of antigen

C. Not tested (skip to 16)

15. Days of negative conversion since confirmation of infection: ____ days

16. Were you home quarantined after infection?

A. Yes (days of quarantine: ____ days)

B. No

**Ⅲ Contact history**

If you were infected, the survey period was in the past 14 days before infection;

If you were not infected, the survey period was from December 2022 to the present.

1. Have you ever contacted with positive family members?

A. Yes

B. No

2. Have you ever contacted with positive patients in the hospital?

A. Yes

B. No

3. Have you ever contacted with positive colleagues in the hospital?

A. Yes

B. No

4. Have you ever contacted with the equipment or items used by positive patients in the hospital?

A. Yes

B. No

5. Have you ever performed aerosol-generating procedures (tracheal intubation, sputum aspiration, nucleic acid sampling, bronchoscopy, etc) for suspected or confirmed patients?

A. Yes

B. No

6. Have you ever contacted with positive person outside the hospital?

A. Yes

B. No

7. Work category:

A. Firstline work (with patient facing)

B. Second line work (without patient facing) (skip to 14)

8. Total time of patient contact every day:

A. <4 hours

B. 4-8 hours

C. >=8 hours

9. Contact time per patient:

A. <5 minutes

B. 5-15 minutes

C. 16-30 minutes

D. >=30 minutes

10. Contact distance from patient:

A. Touched patient

B. 1 meter

C. 2 meters

D. No contact but in the same room

11. Use of personal protective equipment on duty: (multiple choices)

A. Head mounted N95 mask

B. Ear loop KN95 mask

C. Common ear loop surgical mask

D. Lace-up surgical mask

E. Isolation gown

F. Protective clothing

G. Disposable hat

H. Disposable gloves

I. Protective face shield/eye mask

J. Shoe cover

12.Did you have the following oral-nasal exposures on duty? (multiple choices)

A. Taking off the mask during eating in the presence of colleagues

B. Taking off the mask during breaks in the presence of colleagues

C. Taking off the mask when talking with colleagues

D. Improper wearing of mask when contact with patients

E. None

13. Duration of oral-nasal exposures every day on duty:

A. <1 hours

B. 1-2 hours

C. >=2 hours

14. What do you think are the possible reasons of infection? (multiple choices) (skip this question if you were not infected)

A. Contact with positive patients

B. Contact with positive colleagues

C. Contact with positive family members

D. Contact with the equipment or items used by positive patients

E. Exposure to contaminated environment

F. Contact with positive person outside the hospital

G. Inadequate PPE

H. Improper usage of PPE

I. Poor immunity

J. Unknown cause

K. Others________________

**Ⅳ Previous medical history**

1. Do you have underlying diseases?

A. Yes

B. No (skip to 3)

2. What underlying diseases do you have? (multiple choices)

A. Hypertension

B. Diabetes

C. Hyperlipidemia

D. Heart disease

E. Cerebrovascular disease

F. Chronic bronchitis

G. Chronic obstructive pulmonary disease

H. Chronic kidney disease

I. Malignant tumor

J. Immunodeficiency disease

K. Others________________

3. Have you ever been infected with COVID-19 three months ago?

A. Yes

B. No (skip to Ⅴ)

4.Date of infection: yyyy-mm

**Ⅴ Vaccination status**

1. How many doses of COVID-19 vaccine have you received?

A. 0 dose (never vaccinated) (skip to the end)

B. 1 dose (skip to 3)

C. 2 doses

D. 3 doses

E. 4 doses

2. Have you participated in the COVID-19 booster vaccination organized by our hospital at the end of December 2022?

A. Yes (skip to the end)

B. No

3. Whether the last dose of vaccination is more than 6 months from now?

A. Yes

B. No

Thank you again for your support to our study!
